# Targeted self-management limits fatigue for women undergoing radiotherapy for early breast cancer: results from the ACTIVE randomised feasibility trial

**DOI:** 10.1007/s00520-021-06360-0

**Published:** 2021-07-23

**Authors:** Nick Courtier, Jo Armes, Andrew Smith, Lesley Radley, Jane B. Hopkinson

**Affiliations:** 1 School of Healthcare Sciences, Cardiff University, Eastgate House, 35—43 Newport Road, Cardiff, CF24 0AB UK; 2 School of Health Sciences, Faculty of Health and Medical Sciences, Duke of Kent Building, University of Surrey, Guildford, GU2 7XH UK; 3 Centre for Occupational and Health Psychology, Cardiff University, 63 Park Place, Cardiff, CF10 3AS UK; 4 Patient Public Representative, Wales, UK

**Keywords:** Behavioural, Targeted intervention, Prediction, Cancer-related fatigue

## Abstract

**Purpose:**

The ACTIVE intervention uses a novel fatigue propensity tool to target a behavioural fatigue self-management programme for women undergoing radiotherapy for early breast cancer. We assess feasibility and outcomes for ACTIVE.

**Methods:**

Mixed methods comprised a randomised feasibility trial with qualitative process evaluation and a nested fatigue risk substudy. Participants at a higher risk of fatigue were allocated 2:1 to behavioural intervention or information alone. Participants at a lower risk of fatigue entered the fatigue risk substudy. Feasibility was assessed by rates of eligibility, recruitment, retention and adherence. Qualitative interviews explored acceptability of the intervention and trial processes. Measures of fatigue, anxiety, depression, quality of life and self-efficacy were self-reported before, during and 10 days, 3 weeks and 6 months after radiotherapy. Pre-treatment fatigue risk score and post-treatment fatigue were correlated.

**Results:**

Fifty percent (*n* = 75) of eligible patients were recruited with 33 higher risk participants randomised to the trial and 42 entering the fatigue risk score substudy. Trial design and methods were feasible and acceptable with 91% of participants completing all measures according to protocol. Fatigue was clinically-significantly lower in the intervention group during, and in the weeks after, treatment compared to the control: all secondary measures favoured the intervention group. Positive group differences were not maintained at 6 months.

**Conclusion:**

Our targeted approach to fatigue self-management is feasible and acceptable within the early breast cancer pathway. Multiple benefits were reported by patients who received the intervention, which is worthy of further investigation.

**Trial registration:**

ISRCTN 10303368. Registered August 2017. Health and Care Research Wales Clinical Trial Portfolio Registration 31419.

## Introduction

Fatigue management ranks as the fourth most important research question for those living with and beyond cancer [1], reflecting the impact cancer-related fatigue (CRF) has on daily functioning and identity [2, 3]. Fatigue is a substantial issue in Westernised early breast cancer (EBC) care as a function of age standardised incidence rates circa 140 per 100,000, 5-year survival rates between 80 and 90% [4] and an estimated CRF prevalence between 60 and 95% during treatment [5] and 27% after treatment [6].

Physical activity and cognitive-behavioural therapy (CBT) are both recommended interventions for CRF [7, 8]. An effective fatigue intervention for EBC populations would maintain function and reduce sedentary behaviours associated with excess cancer recurrence and secondary health complications in post-menopausal women [9]. Combining activity with skills to self-manage thoughts/behaviour is important for women with EBC undergoing adjuvant treatment, where variance in fatigue correlates with mood [10]. Motivation is also key, as adherence to behavioural interventions is difficult when suffering fatigue.

Nearly all women with EBC receive adjuvant radiotherapy as their final treatment before self-managed survivorship. Those with poorer prognostic factors are prescribed months of adjuvant chemotherapy prior to radiotherapy, creating two bio-behaviourally distinct groups. Approximately four-in-ten people receiving radiotherapy for breast cancer suffer fatigue at a level that significantly disrupts functioning [11]. The remaining 60% appear to be little affected prior, during and after radiotherapy [11, 12]. As elevated fatigue at the end of radiotherapy treatment is associated with chronic fatigue [6, 13], the optimal management scenario would be to identify women at risk of fatigue sufficiently early to limit its development. Yet, the majority of fatigue management interventions have been tested in patients undergoing chemotherapy, when fatigue has become established [14] or include patients unlikely to derive benefit due to low fatigue propensity [15]. We have developed ACTIVE, a behavioural intervention that comprises a fatigue propensity tool [12] and a fatigue self-management programme. The current study uses the propensity tool to predict patients at a lower risk of experiencing fatigue during radical radiotherapy for EBC: these participants enter a propensity tool validation substudy. Participants predicted to be at a higher risk of fatigue enter a trial of the ACTIVE self-management intervention. Study objectives were to (i) determine if the trial design is feasible to deliver in the radiotherapy pathway, (ii) evaluate the acceptability of the intervention and trial processes and (iii) estimate unknown parameters for an effectiveness trial.

## Methods

### Design

Feasibility of the ACTIVE intervention was tested by a parallel phase II randomised controlled trial (ISRCTN 10,303,368) and reported following CONSORT guidance [16]. A process evaluation comprising qualitative interviews sought to understand the acceptability of the intervention and trial processes for design refinement purposes. The full trial protocol [17] was approved by NHS Research Ethics Committee16/WA/0205.

### Setting and participants

The intervention was delivered at a regional cancer centre in the UK and evaluated by patient-reported measures. Patients were eligible if they were female > 16 years, stage 0–IIIA breast carcinoma, prescribed standard 40 Gy in 15 fractions over 3 weeks ± nodal or tumour bed irradiation. Ineligibility was defined by palliative intent, concurrent chemotherapy, serious comorbidity causing fatigue, psychiatric illness requiring secondary care, too ill to engage and participant in other trial.

### Recruitment procedure

The radiographer-led team screened patients scheduled for a treatment planning scan. Team members approached eligible patients after their scan and introduced those interested to a researcher for written and verbal information. Patients then indicated their willingness to participate via a postal slip or a follow-up telephone call.

### Fatigue risk score

**Fig. 1:**
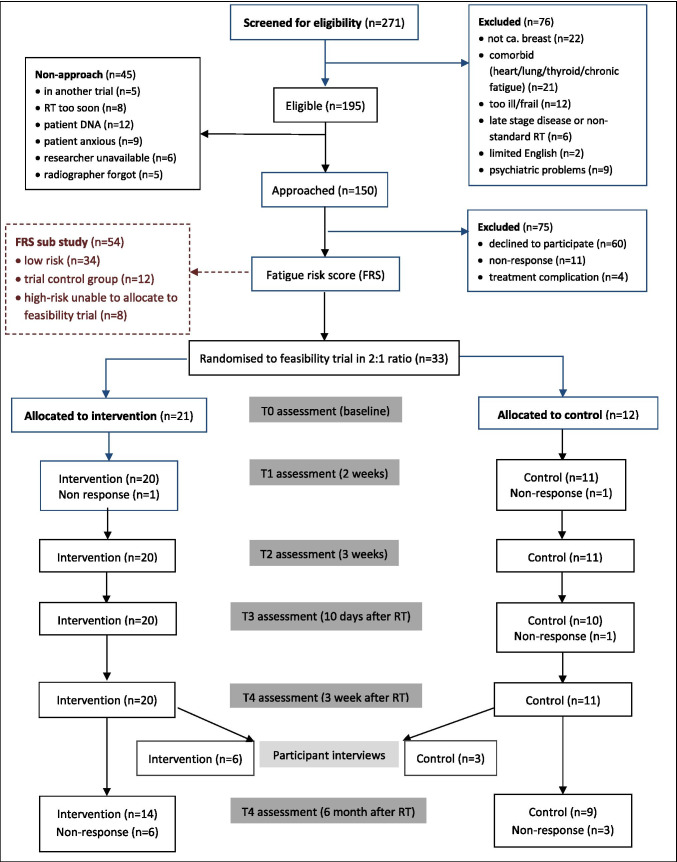
Flow plan of participants through study

Interested women were telephoned to address queries, confirm willingness to participate and calculate a fatigue risk score (FRS). The FRS tool dichotomises patients at a higher/low risk of experiencing fatigue during radiotherapy [12]. FRS ≥ 5 (higher risk) initiated allocation into the trial. Participants with FRS < 5 (insufficient risk for intervention) were included in a ‘FRS validation substudy’ (Fig. 1.)

### Sample size

Assuming 10% attrition, we aimed to recruit 33 participants for a final trial sample of 30. The target sample size was informed by pooled effect size estimates for non-pharmacological fatigue interventions [7] and guiding literature [18, 19]. Based on previous experience [10] we assumed 45% of participants would have FRS ≥ 5 and a recruitment rate of 50%, suggesting 150 eligible women would need to be approached to achieve the target sample.

### Trial consent and randomisation

Written consent had to be given within 10 days of invitation to enable group allocation before radiotherapy. Participant characteristics and baseline measures were recorded immediately after consent. Allocation in the ratio 2:1 to the ACTIVE intervention or control group was via a remote online database [20] using a permuted block protocol. The minimisation variable < or ≥ 57 years aimed to balance groups around the population mean. Only outcome assessors were blinded to group allocation.

### Trial interventions

An ‘information alone’ comparator was included to assess acceptability of randomisation and to understand the importance of interactions with intervention providers. This control group were given the Macmillan Cancer Support ‘Coping with Fatigue’ booklet [21] that was freely available at the centre. Full details of the ACTIVE intervention have been published [17]. In brief, it consists of education about fatigue, motivation to be active, goal setting and behavioural regulation and emotional support. A Fitbit Alta™ activity tracker was provided to be worn for 14 days. The intervention was delivered in three sessions: at the start of radiotherapy, after ten treatments and at the end of the treatment. The intervention was grounded within a CBT model of symptom management [22] with motivational interviewing elements to encourage goals that were meaningful for individuals [23]. The 60-min sessions were delivered face-face by a professional attached to the centre psychology team. Sessions were audio-recorded for quality assurance purposes. An independent assessor evaluated the first three sessions plus a random selection for intervention fidelity and the use of underlying theory.

### Trial outcomes

Feasibility.

Rates of eligibility, invitation, recruitment, allocation, retention, adherence and adverse events were calculated. Ethical approval allowed reasons for decline/discontinuation to be sought. A follow-on multi-centre effectiveness trial was considered feasible if > 70% of participants completed all interventions and outcome measures. A 65–70% rate would require adjustments to the intervention/trial processes to proceed, whilst < 65% required substantive change.

#### Acceptability

A sample of participants with varying engagement and responses to the intervention were purposively selected to explore their views and experiences of the trial in semi-structured interviews. Interviews were also undertaken with intervention providers and control group participants to explore acceptability of, and impacts from, participation.

### Trial measures

Measures (Table 1) were all self-reported validated questionnaires [17]. The primary endpoint was median fatigue measured by the Functional Assessment of Chronic Illness Therapy Fatigue Scale (FACIT-F) [24] at 10 days after radiotherapy, when fatigue was assumed to peaks.

**Table 1:** Schedule of trial outcome measures

Outcome measure	Pre-RT	Week 2	Week 3	+ 10 days	+ 3 weeks	+ 6 months
	Before RT treatment	After 10 RT treatments	End of RT treatment	10 days after RT	3 weeks after RT	6 months after RT
	T0	T1	T2	T3	T4	T5
Fatigue	X	X	X	X	X	X
Anxiety	X			X		X
Depression	X			X		X
QoL	X		X	X		X
Self-efficacy	X		X	X		X

### Data analysis

Feasibility statistics were described with 95% confidence intervals. Mean/median point statistics for longitudinal measures were compared between groups on an intention-to-treat basis. Effect size was estimated for the primary endpoint. Interviews were analysed using the framework approach [25]. A coding frame for generated themes was developed and validated with half the data being double-coded. The predictive performance of the FRS was assessed, for the FRS substudy and all trial participants who did not receive the intervention, by its correlation with FACIT-F score at 15 fractions and 10 days after radiotherapy.

## Results

The study addressed three research objectives.

### *Objective 1*—Feasibility of trial design

#### Recruitment

One hundred ninety-five of 271 patients screened between 22 July 2016 and 23 June 2017 met study eligibility criteria: an eligibility rate of 72% (CI_95%_ = 66–77%). Comorbidities and being too ill/frail were common ineligibility characteristics. Forty-five (23%) of eligible patients were not invited for logistical reasons (Fig. 1). Seventy-five of the remaining 150 patients agreed to participate, a recruitment rate of 50% (CI_95%_ = 41–58%). Common reasons for declining were ‘too complicated/too much to think about/too busy/don’t want additional appointments’. Only 22 people opted to indicate their interest using a reply slip (11 returned.) Posted slips were also logistically problematic, as they had to be received in time for group allocation.

#### Trial groups

Thirty-four (45%) and 41 (55%) of participants were predicted to be at a low and high risk of fatigue respectively. It was not possible to allocate eight of the high-risk participants to the trial whilst the (then lone) intervention provider was unexpectedly not available for a period of six weeks. Fatigue data from these participants was instead entered into the FRS validation substudy. The final trial sample size of 33 exceeded the target, with 21 in the intervention and 12 in the control group (Fig. 1). Clinical characteristics of trial participants (Table 2) were representative of the breast cancer population at the study centre. The intervention group were, by chance, more likely to have received prior chemotherapy than the control group, being, on average, younger with a higher proportion of grade 3 disease.

**Table 2:** Baseline demographic and clinical characteristics of feasibility trial participants

	Intervention (*n* = 21)	Control (*n* = 12)	All (*n* = 33)
	Mean (SD)		
Age (years)	57.4 (10.2)	60.1 (7.5)	58.3 (10)
Body mass index (kg/m^2^)	29.2 (5.3)	27.6 (5.6)	28.6 (5.4)
Index of multiple deprivation	1212.8 (539.4)	1023.6 (601.5)	1143.9 (552.8)
Time from surgery to radiotherapy (days)	61.5 (12.2)	61.0 (16.7)	61.3 (13.7)
	Frequency (% of group)		
Work			
Retired/housewife Changed/postponed Continued work	5 (24) 14 (66) 2 (10)	7 (58) 4 (34) 1 (8)	12 (36) 18 (55) 3 (9)
Diagnosis			
Carcinoma in situ Invasive ductal cancer Invasive lobular cancer	2 (10) 15 (71) 4 (19)	1 (8) 8 (67) 3 (25)	3 (9) 23 (70) 7 (21)
Tumour/Node/Metastasis stage group			
0 I II III	2 (10) 6 (29) 10 (48) 3 (13)	1 (8) 5 (41) 5 (42) 1 (8)	3 (9) 11 (33) 15 (46) 4 (12)
Laterality			
Right Left	13 (62) 8 (38)	5 (42) 7 (58)	18 (54) 15 (46)
Grade			
1 2 3	1 (5) 9 (43) 11 (52)	2 (17) 6 (50) 4 (33)	3 (9) 15 (45.5) 15 (45.5)
Surgical procedure			
Wide local excision Mastectomy	19 (91) 2 (9)	11 (92) 1 (8)	29 (91) 3 (9)
Chemotherapy			
Yes No	14 (67) 7 (33)	5 (42) 7 (58)	19 (59.4) 13 (40.6)
Travel mode for treatment			
Self Hospital transport	21 (100) 0	12 (100) 0	33 (100) 0
Breast boost 10 Gy/5#/1 week	1 (5)	1 (8)	1 (6)
40 Gy/15#/3 weeks to SCF	1 (5)	0	1 (5)

#### Trial retention and adherence to protocol

One participant dropped out of each trial group before start of treatment with no reasons proffered: a trial retention rate of 94%. The remaining 20 in the ACTIVE group returned all measures, whilst one control group participant forgot questionnaires at T3. Ninety-one percent (CI_95%_ = 81–100%) of trial participants completed all the interventions and study measures according to protocol, surpassing the > 70% criterion for feasibility. This statistic excluded 6-month follow-up measures, which were returned by 14/20 (70%) and 9/11 (82%) of intervention and control groups respectively: an overall 6-month response rate of 74%.

### Objective 2—Acceptability of trial process and intervention

#### Trial process

Interview data confirmed the acceptability of trial processes. Longitudinal measures were considered relevant, timely and not onerous:

*The points at which the questionnaires had to be completed were very important points. They let me evaluate my response to the treatment.* [Control2005]

*The paperwork was spread out so I didn’t have to do it all in one go when I was tired.* [Intervention1095]

One participant found the questions *‘strange’* at first but the meaning gained with hindsight suggested the questionnaires were performing as part of the intervention:

*You think you’re getting over it but it actually starts getting worse. So I now understand why the questions are there. I thought, ‘this is what they’ve been talking about’, but you don’t know until it hits you.* [Intervention1071]

One control participant commented on the repetitive nature of the measures, wondering *‘if they were trying to catch you out by asking the same question?’.*

Being labelled at a ‘high risk’ of fatigue could motivate or engender surprise or transient worry. Two of the three interviewed control group participants revealed a preference for allocation to the intervention group:

*I would have found it interesting talking with the counsellor.* [Control2055]

*It would have been nice that somebody else was keeping an eye on me and getting me going.* [Control1053]

Control group participation had perceived benefits in terms of self-monitoring response to treatment and putting into practice the *‘common sense’* information in the booklet.

#### Intervention fidelity and adverse effects

All intervention sessions were delivered within 4 days of schedule. Three of the final sessions were delivered by telephone due to changes to radiotherapy appointments. The intervention was suspended for one participant whilst they accessed psychological support. A case referral form was consequently introduced with augmented psychological history. One subsequent participant was offered a separate referral for underlying psychological issues but opted to continue with ACTIVE. Two participants reported minor skin reactions from the Fitbit strap. A more common technical problem encountered by participants was uploading Fitbit data. An interim decision was made not to pursue remote access of data by the researchers, but to continue to follow the principal protocol aim of investigating if/how participants chose to use the device.

#### Acceptability of intervention

Interviews corroborated the high degree of satisfaction with study processes suggested by retention data. Emergent themes are summarised in Table 3. After some initial intrigue/scepticism that fatigue could be managed, negative comments were limited to confusion regarding when an activity plan should be completed and *‘not wanting to hang around having had radiotherapy’.* One respondent questioned whether, after a rapport-building session, the next two could be by phone or online. Provider interviews provided insight about a lack of flexibility when coordinating intervention appointments between participants and a planned but unpredictable radiotherapy schedule.

**Table 3:** Themes developed from participant interviews

Theme	Illustrative quotes
Normalisation of fatigue … and rarely mentioned emotional and psychological effects. A sense of control facilitated by evaluating response to treatment	… it was comforting just to know that it’s okay, it’s normal and it will improve. You feel like you’re part of this big family. (1093) A better understanding of how tired I was feeling and how I was feeling about how tired I was feeling, and what I could do to manage my expectations or how I felt, that was the thing. You think that fatigue is a physical issue don’t you, but when you dig deeper I don’t think it is. It comes with the emotional side as well and the mood and I suppose there were broader issues really, but it was like I was trying to deal with it too head-on. And then when I was in the sessions it made me realise that perhaps it wasn’t just the physical side that struck me down. (1081) By writing and thinking, ‘Well, hang on, last time I was this but now I’m this’, you could see the difference because it messes with your memory and you can’t remember every day anyway. (1071) The intervention was brilliant because I was able to speak and look at coping mechanisms. It was a pattern, so perhaps when I did have energy and perhaps when I was feeling very tired, obviously being able to relate back to the diary then you could put coping mechanisms into place there. (1081)
Technology motivates Activity tracker overcomes resistance to activity fear of fatigue, and sedentary behaviours	I loved having that active thing on my wrist. That was brilliant, I didn’t want to give that back. Because it made me – it challenged me to do more, to have a look at it and have a go, because I’d set it up on my tablet. Every day I’d have a look and “Wow, did I do that?” So that was like a challenge. (1114) The tracker made me go, “I’m going to do this many steps today”, but I only did that if I felt well enough to do it. But I always went out anyway. So that was an incentive to fight the risk of fatigue. (1071) There were times when it was reminding me to move, and I’d go, I don’t want to, but I think that is normal. I mean there were times when you’re sitting around waiting for the radiotherapy and travelling back there was a good 2 ½ hours when I really wasn’t able to be very active. Other than that, it did make me conscious of how long it had been since I had moved. There was at least one occasion where I went over 8000 and it had a little party on there! Subjectively I suppose it, it doesn’t feel like it’s getting any easier but if you go for a walk or a run or whatever but then, it’s nice to have something that tells you that you are going slightly faster than you were before or further, that kind of thing really. (1093)
Caring for self Opportunity to prioritise self makes for a restorative effect	Just keep the one-to-one intervention because it was so refreshing to be able to talk to somebody that wasn’t directly connected to the treatment, but was in that environment. To have a dedicated session about things that might be worrying me around my activity levels. I suppose it gave me an opportunity to reflect more on what I was thinking and feeling, and how I was behaving. Although I am not sure it was always related to fatigue. (1060) Opening up, being able to say how you felt and I probably opened up too much, because I think I started talking about my life a little bit! But she was an amazing listener, that’s so important to have the right person to ask those questions. She was really brilliant, independent from the nurses—kind of like a friend. So yeah, it was far better than what I thought it was going to be, like I’ve mentioned the coping mechanisms that was needed to push myself that little bit further (1071) I would have just carried on doing as much as I could anyway but it made me stop and think about resting as well because I don’t always get the rest that I need (1114) I didn’t have a lot of enthusiasm for anything when I was going through this period. So it helped me focus on good things and get on with some of the activities that I enjoyed in the past, and had stopped doing while I was going through this treatment. (1060)
Importance of identity Activity helps maintain self-identity when tailored to contexts	You’re feeling so tired and you feel you’ve lost, do you understand what I’m saying? You’re not being able to fulfil activities and things like that and I felt, not sure useless is the right word really but everything is an effort. Not feeling emotional just you’ve got heavy energy: probably useless is a good word to be honest. (1081) I did find I had a bit of strange obsession with ironing! Not that I did that much, but it was really agitating me that I wasn’t doing it. It is difficult to analyse yourself … I said, ‘I’m not going to give in to it’, because you can fight your way through it. If I sat down, I thought, “Oh God, I don’t want to get up”, but then we thought ‘No, I’m going to get up and I’m not going to let it beat me.’ (1093) When I returned to work I was surprised how tired I felt but we’d gone through some ways that can be managed. I’m still trying to follow that pattern of good health. I thought it was good that they had these exercise sessions geared towards people with cancer and I don’t think they do them locally but I know there’s an organisation in Y. (1060)

Intervention providers raised thematically similar points. Confirmation that people were on the right track in managing their fatigue gave confidence to focus the CBT aspects of the intervention on people’s goals and self-care, which importantly engendered a feeling of ‘moving on in life’. It was seen as important to acknowledge the ‘*rippled effect*’ of fatigue, impacting on many life domains, but this created tension at the boundaries of the intervention. One of the providers noted that a skilled interventionist was needed to manage participant distress from functional limitations without producing an emotionally-charged therapy session:

*I did find myself having to balance not doing any harm therapeutically by closing patients down when they would voice their concerns and distress about their ability not being able to do the things that they used to do and also delivering the intervention, as a proactive way to manage that and resume some level of activity.*

Review of session transcripts demonstrated good fidelity to the theoretical basis of the intervention, with frequent use of education, goals and behavioural regulation, moderate use of intentional reinforcement and optimism and some boosting of self-efficacy and motivation. The participant selection was questioned as some of those receiving the intervention turned out to have a greater level of resilience and activity than some patients from the intervention provider’s clinical experience. All participants were perceived to derive benefit from the intervention, but excluded patients may have gained more.

### Objective 3—Estimation of unknown parameters

#### Between-group comparisons of trial mseasures

Groups were comparable at baseline on all outcome measures (Table 4.) Cohen’s *d* effect size for the primary endpoint of mean fatigue measured by the FACIT-F at T3 was estimated as 0.3 (CI_95%_-0.41–1.01). Fatigue was lower in the intervention group during and at 10 days and 3 weeks after treatment. However, 6-month follow-up data indicated lower mean and median fatigue in the control group. Secondary outcomes favoured the intervention group during treatment and 3 weeks after, with comparable data at 6 months.

**Table 4:** Longitudinal outcome measures described by trial group

	T0 Baseline		T1 Week 2	T2 Week 3	T3 + 10 days	T4 + 3 weeks	T5 + 6 months
Measure	Mean (SD) 95% confidence interval Median (IQR)						
FACIT-F							
Intervention		19.3 (8.8) 15.1–23.6 20 (9)	20.0 (11.5) 14.8–25.6 18 (17)	18.6 (9.9) 13.8–23.3 18 (14)	18.8 (9.9) 14–23.6 18 (16)	17.8 (10.5) 12.8–22.9 18 (17)	17.1 (9.4) 10.4–23.8 17 (17)
Control		19.6 (10.2) 12.3–26.9 19 (15)	19.8 (9.1) 13.2–25.5 23 (17)	21.0 (9.7) 14.4–27.5 25 (16)	21.7 (10.7) 14.6–27.1 25 (25)	19.3 (10.2) 12.4–26.1 26 (15)	9.1 (9.0) 1.6–16.7 6 (18)
QLQ30							
Intervention		8.6 (2.0) 7.6–9.6 8 (3)		8.8 (2.5) 7.6–9.9 8 (2)	8.8 (2.8) 7.5–10.1 8 (3)		8.5 (2.6) 6.7–10.4 8 (3)
Control		9.8 (4.2) 7.1–12.4 9 (7)		8.3 (2.7) 6.5–10.1 8 (4)	9.2 (3.5) 6.8–11.6 8 (5)		7.3 (2.8) 4.9–9.6 6 (7)
AH self-efficacy							
Intervention	34.8 (12.9) 28.6–41.1 35 (17)			40.8 (10.2) 35.9–45.7 41 (18)	42.2 (10.7) 37.1–47.3 46 (19)		
Control	34.7 (7.3) 30.1–39.2 34 (11)			38.8 (14.4) 29.1–48.5 39 (20)	35.3(17.5) 23.5–47.1 30 (22)		
HADS anxiety							
Intervention	7.7 (4.2) 5.7–9.7 8 (4)				6.6 (4.0) 4.6–8.5 6 (4)		5 (3.9) 2.2–7.8 4 (8)
Control	7.9 (3.1) 6.0–9.9 8 (6)				8.5 (4.1) 5.7–11.2 9 (6)		5.3 (4.7) 1.3–9.2 4.5 (6)
HADS depression							
Intervention	5.8 (3.5) 4.2–7.5 6 (5)				5.6 (3.7) 3.9–7.4 5 (5)		5.6 (3.7) 3–8.2 5 (4)
Control	5.6 (3.7) 3.3–7.9 6 (6)				6.7 (3.7) 4.2–9.2 7 (8)		3.9 (2.1) 2.1–5.6 3.5 (3)

#### Performance of fatigue risk score

The relationship between the pre-treatment FRS and fatigue after 15 fractions and 10 days after radiotherapy was explored by Spearman correlations. The FRS validation group was *N* = 54, but missing data on FACIT-F scores at one of the time points meant that all analyses were *N* = 50 (Table 5.) There were no statistically significant differences for those that had/had not received chemotherapy (*z* =  − 0.31, *p* = 0.76 two-tailed). A full validation of the FRS accuracy will be presented separately.

**Table 5:** Correlations of fatigue risk score and fatigue after 15 fractions and 10 days post-RT

		Whole group *N* = 50	No chemo *N* = 27	Chemo *N* = 23
FRS v FACIT fatigue at 15#	*ρ*	0.81	0.81	0.84
	95%CI^*^	0.65 to 0.91	0.53 to 0.96	0.64 to 0.95
	*p*	< 0.001	< 0.001	< 0.001
FRS v FACIT fatigue at + 10 days	*ρ*	0.74	0.79	0.75
	95%CI^*^	0.61 to 0.84	0.61 to 0.9	0.47 to 0.89
	*p*	< 0.001	< 0.001	< 0.001

^*^Bootstrapped CI

*FRS,* fatigue risk score; *FACIT-F*, Functional Assessment of Chronic Illness Therapy Fatigue Scale

## Discussion

This trial has successfully established feasibility and acceptability of a targeted behavioural intervention to minimise fatigue in people with EBC. Outcomes for those receiving the ACTIVE intervention compared favourably with receiving education alone at the end of radiotherapy, a crucial point marking the start of self-managed survivorship for this population [26].

### Feasibility

The trial design and methods are feasible (lower bound of 95% confidence for feasibility measure exceeding pre-specified threshold.) Targets for recruitment and randomisation were met. Greater recruitment and intervention delivery capacity within a multi-centre design hold potential for further improving the recruitment rate. Intervention provider interview data suggested the comorbidity exclusion criteria could be relaxed to include more women that would benefit from the intervention: The downside being a weakening of internal validity due to the inclusion of people experiencing fatigue unrelated to their cancer treatment. Terminology used at first contact was seen to be able to restrain participation for those feeling least able to cope with their life predicament (excellent candidates for ACTIVE): not therapeutically loaded ‘counsellors’ to deliver the intervention and not study ‘groups’, which could imply an unwanted group intervention. Both intervention providers (a radiographer and a counsellor) had prior counselling training. Our manual-based approach with associated training and monitoring of intervention fidelity provides clear indications of how to support intervention delivery by a variety of trained health professionals, particularly radiotherapy staff.

### Acceptability of process and intervention

High adherence and low attrition rates were corroborated by positive accounts of both ACTIVE and trial processes. Offering the intervention to those above a pre-treatment fatigue risk represents a novel application of a targeted secondary preventive intervention. That is, prophylactic use to prevent worsening of an existing or latent problem: an idea much in vogue during the COVID-19 pandemic. Focussing on the acceptability (rather than performance) of the FRS score, the high/low fatigue risk dichotomy held mixed valence for our participants. Being categorised at a ‘higher risk of fatigue’ was a potent motivator for change but a minority found this unsettling or stigmatising. A range of responses is congruent with theories of targeted interventions applied to models of behaviour that focus on health risk perception [27, 28]. This aspect warrants further refinement that emphasises predictions may not be realised.

Interview data helped understand how ACTIVE normalised fatigue and enhanced a sense of control and motivation towards activity; important cognitive precursors of positive behaviours [29]. The Fitbit tracker proved a persuasive tool to overcome resistance to both activity and fears surrounding the impact of fatigue on daily living. The benefit of instant physiological feedback supporting self-efficacy reported in non-cancer populations [30, 31] was evident here. The device also raised awareness of the sedentary behaviour associated with cancer and secondary health complications [9, 32–34]. Conversely, it could help manage over-exertion, or fears of this. The net effect was to bolster intentions to maintain longer term activity behaviours, a key problem reported for breast cancer survivors [35]. The efficient upload of physiological data will be important to realise in a future trial.

Prioritising self-care and enjoyable activities were as important as increasing physical activity, in terms of engendering a restorative effect that was both intrinsically valuable and has been linked with maintenance of activity [36]. Intervention providers felt that more space was required for work that explored the underpinning emotional and psychological rationale behind people’s behaviours. However, this would act against the delivery of a time-limited intervention during a standard course of radiotherapy.

### Longitudinal measures by trial group

The patient-reported outcomes support proceeding to follow-on work with a larger sample size. As assumed a priori, the course of median fatigue increased for the control group during RT but reduced slightly for the intervention group. There was a difference of seven points in the primary endpoint of median FACIT-F fatigue at 10 days after radiotherapy (3 scale points define a clinically significance change) [37]. The other outcome measures—quality of life, self-efficacy, anxiety and depression—also all favoured the intervention group at this point. Most patients would be expected to recover (to some extent) in the months post-treatment, with the intervention group having relatively less of a recovery to make. However, the expected positive relationship between fatigue at end of treatment and 6-month follow-up was reversed here, with a clinically significant lower fatigue level reported by the control group. One partial explanation is suggested by patient interviews indicating that study questionnaires and information booklet had stimulated the control group to adopt strategies to manage their fatigue. Secondly, despite randomisation, the intervention group were slightly younger with slightly poorer prognostic factors relative to the control group. This meant that prior chemotherapy was more likely and more intensive treatment may plausibly have hindered recovery: for example, a greater proportion of the intervention group stopped working during treatment, potentially impairing physical or emotional health. The role of chemotherapy prescription on fatigue and mood data was not supported by exploratory *t*-tests. Missing data could also partly explain the anomalous 6-month fatigue pattern. The 30% of the ACTIVE group who did not return questionnaires at follow-up may have considered they no longer had a fatigue problem, or conversely the 18% control group non-returners may have been particularly fatigued. A final explanation is that the intervention only has a short-term effect, needing a booster session after acute effects of radiotherapy recede.

We acknowledge inherent limitations in this feasibility trial. An effect size has been estimated to guide a future sample size calculation, but the small sample, and hence wide CI, mean this figure should be interpreted cautiously against relevant published evidence [7]. The complex group allocation may have underestimated the effectiveness of ACTIVE by excluding women who would have benefited most. For example, patients using hospital transport were effectively excluded might benefit from a hybrid delivery of the first session face-to-face and subsequent sessions online. Finally, despite data analysis being conducted blind to group allocation, bias cannot be ruled out in an open label trial where evaluating the impact of contact with the intervention provider was a feature of the design.

Feasibility objectives were met and results support full-scale effectiveness testing that includes health economic evaluation. Development is indicated to evaluate if radiographers can be trained to deliver the intervention, and the inclusion of an online self-help module is worthy of consideration to maintain support after treatment has completed. The end of radiotherapy is a crucial milestone for patients with early breast cancer as intensive treatment is replaced by open-ended self-management. We have demonstrated how support targeted based on fatigue risk can minimise the development of fatigue by maintaining participation in valued activities and reduction of the sedentary behaviour associated with poorer cancer and wider health outcomes.

## Data Availability

Reasonable requests for the datasets used and/or analysed during the current study will be considered by the ACTIVE TAG.
